# Cry for health: a quantitative evaluation of a hospital-based advocacy intervention for domestic violence and abuse

**DOI:** 10.1186/s12913-019-4621-0

**Published:** 2019-10-21

**Authors:** Gemma Halliwell, Sandi Dheensa, Elisabetta Fenu, Sue K. Jones, Jessica Asato, Suzanne Jacob, Gene Feder

**Affiliations:** 1 Domestic Violence/Abuse and Health Research Group (DVAHG), Centre for Academic Primary Care, Bristol Medical School, University of Bristol, Canynge Hall, 39 Whatley Road, Bristol, BS8 2PS UK; 2 Royal College of Physicians, 11 Saint Andrews Place Regent’s Park, London, NW1 4LE UK; 3 Safelives, Suite 2a, Whitefriars, Lewins Mead, Bristol, BS1 2NT UK

**Keywords:** Domestic violence and abuse, Intimate partner violence, Advocacy, Healthcare response, Accident and emergency (ED)

## Abstract

**Background:**

Domestic violence and abuse (DVA) damages the health of survivors and increases use of healthcare services. We report findings from a multi-site evaluation of hospital-based advocacy services, designed to support survivors attending emergency departments and maternity services.

**Methods:**

Independent Domestic Violence Advisors (IDVA) were co-located in five UK hospitals. Case-level data were collected at T1 (initial referral) and T2 (case closure) from survivors accessing hospital (T1 *N* = 692; T2 *N* = 476) and community IDVA services (T1 *N* = 3544; T2 *N* = 2780), used as a comparator. Measures included indicators of sociodemographic characteristics, experience of abuse, health service use, health and safety outcomes. Multivariate analyses tested for differences in changes in abuse, health and factors influencing safety outcomes. Health service use data in the 6 months pre-and post- intervention were compared to generate potential cost savings by hospital IDVA services.

**Results:**

Hospital IDVAs worked with survivors less visible to community IDVA services and facilitated intervention at an earlier point. Hospital IDVAs received higher referrals from health services and enabled access to a greater number of health resources. Hospital survivors were more likely to report greater reductions in and cessation of abuse. No differences were observed in health outcomes for hospital survivors. The odds of safety increased two-fold if hospital survivors received over five contacts with an IDVA or accessed six or more resources / programmes over a longer period of time. Six months preceding IDVA intervention, hospital survivors cost on average £2463 each in use of health services; community survivors cost £533 each. The cost savings observed among hospital survivors amounted to a total of £2050 per patient per year. This offset the average cost of providing hospital IDVA services.

**Conclusions:**

Hospital IDVAs can identify survivors not visible to other services and promote safety through intensive support and access to resources. The co-location of IDVAs within the hospital encouraged referrals to other health services and wider community agencies. Further research is required to establish the cost-effectiveness of hospital IDVA services, however our findings suggest these services could be an efficient use of health service resources.

## Background

Domestic violence and abuse (DVA) is a public health problem and challenge to clinical services. The estimated cost of DVA to the United Kingdom (UK) is £23 billion per year, including health costs to the NHS of £1.73 billion [1]. DVA can include physical and sexual violence, coercive, controlling and emotionally abusive behaviours, economic restrictions, as well as harassment and stalking. In England and Wales 27% of women and 17% of men report lifetime experience of DVA [2]. Most severe and repeated assaults are directed at women, committed by male partners within the context of an intimate relationship [3].

Intimate partner violence (IPV) can have life-long physical and mental health consequences [4]. The association with mental health problems is strong, with survivors at heightened risk of experiencing anxiety, depression, suicidal behaviours and PTSD [5, 6]. The severe health outcomes of IPV leads to increased use of healthcare services (e.g., primary care, emergency departments (ED), mental health) [7, 8]. As such, healthcare professionals are uniquely positioned to identify and respond to disclosures of abuse and have a crucial role to play in the prevention and management of IPV [9]. However, evidence shows that while 54% of all women presenting at ED are likely to have lifetime experience of IPV, only 5% are identified by healthcare professionals [10].

### The role of healthcare services in addressing intimate partner violence

Guidance from the National Institute for Health Care Excellence (NICE) and the World Health Organisation (WHO) recommends that survivors presenting with indicators of IPV in healthcare settings should be asked about abuse by trained healthcare professionals and support for IPV should be integrated into health settings [11, 12]. Yet, healthcare professionals often face barriers in identifying abuse [13, 14]. This difficulty has been attributed to lack of training, time restrictions, confidentiality and autonomy, attitudes about IPV, as well as discomfort and low confidence. Organisational factors also play a part, including lack of resources and suitable environments for disclosures, absence of policies and protocols about IPV, unclear referral pathways and lack of co-ordination between services [15, 16]. Integration of support for IPV in healthcare settings remains slow and few healthcare-based interventions for IPV have been evaluated systematically, making it difficult for providers, policymakers and researchers to understand how to effectively intervene [13, 14].

### Independent domestic violence advisors (IDVA) within hospital settings

One approach to training healthcare professionals to identify and respond to IPV is to offer survivors on-site DVA advocacy services. Advocacy support for survivors at highest risk of harm or fatality has been formally recognised in the role of the Independent Domestic Violence Advisor (IDVA). IDVAs risk assess, safety plan and help survivors to access services. IDVAs’ work can include advocacy at Multi-Agency Risk Assessment Conferences (MARAC) as well as support with the criminal and civil justice system, emergency housing, health and wellbeing, finances, employment and immigration [17]. IDVA services sit independently of other agencies (e.g., police, refuge) and are designed to be delivered from the point of crisis over a relatively short period of time, before referral onwards to longer-term community services (e.g., outreach services). Meta-analyses show that advocacy for severe IPV can improve quality of life and reduce physical abuse short-term, but there is limited evidence of its impact on mental health outcomes [18]. Despite the increased likelihood of survivors attending ED for the health-related consequences of IPV, few studies have evaluated the role of advocacy in hospitals. Hyman [19] reported outcomes of a short advocacy intervention (90 min, one contact with patient) delivered in ED. Findings suggested advocacy reduced psychological distress at three to four-month follow-up, but not PTSD symptomology. Two studies exploring the efficacy of hospital IDVA services (located in gynaecology, genitourinary medicine [GUM], HIV clinics and ED) reported an increase in detection rates of DVA among survivors, higher referrals to hospital IDVAs from health services and improvements in healthcare professionals’ confidence in tackling the issue [20, 21]. However, existing studies have reported findings from single sites (e.g., one hospital) across varying healthcare contexts and lack outcomes reported by survivors, cost analyses or follow-up data.

### Aim

We present findings from a multi-site evaluation of an advocacy approach to supporting survivors of DVA in a hospital setting, exploring its impact on improving access to support, health outcomes and cost effectiveness. Independent Domestic Violence Advisors (IDVAs) were co-located within ED and maternity services in hospitals across the UK during April 2012 to November 2015.

The key components of the role included: providing immediate support and advice, risk assessment and safety planning; referral into external services; partnership-work with hospital departments and community agencies; and training hospital staff about DVA. The evaluation sought to answer the following research questions:

1. Who are the survivors accessing help through hospital IDVA services (compared to community IDVA services)?
2. What do hospital IDVAs do (compared to those based in the community)?
3. What impact on survivors’ risk, safety, health and wellbeing do hospital IDVAs have (compared to community IDVAs)?
4. What is the cost effectiveness of hospital IDVA services?

This article presents analysis of quantitative datasets reported by the original research ‘A Cry for Health: Why we must invest in domestic abuse services in hospitals [22]. A comparison group of survivors from community IDVA services was employed to test differences between outcomes for community and hospital IDVA services.

### Data collection

Individuals accessing IDVA services were eligible for inclusion in the sample if they wished to receive support from an IDVA and had consented to the use of their anonymised information for monitoring or research purposes. There were five hospital IDVA services who participated and five community IDVA services based within specialist DVA agencies. Two datasets reflect case level data collected from individuals who had accessed either service.

*Case-level dataset* contained data relating to the geographical areas covered by the IDVA services. Data were collected at two time points. Time 1 reflected intake at IDVA services (T1 *N* = 692 hospital; *N* = 3544 community). T2 reflected the point at which there was a planned case closure or when the client exited a service (*N* = 476 hospital; *N* = 2780 community). Time 2 data were available for 68.7% of the Time 1 hospital IDVA sample and 78.4% of the community IDVA sample.

*Sociodemographic and referral information.* At Time 1 information was collected on participants’ gender, age, sexual orientation, ethnicity, household income, pregnancy, children in the household, relationship status, living arrangements. Information collected around the complex needs and additional vulnerabilities of participants related to mental health concerns, substance use, financial issues and disability. Referral information included items such as who the original referrer was, from which hospital department and the original presenting issue. Referral forms were collected at Time 1, for hospital survivors only (T1 *N* = 198).

*Safety.* Data pertaining to DVA was recorded by IDVAs at T1 (abuse occurring within the previous 3 months) and T2 (abuse experienced during the intervention period). The Severity of Abuse Grid is an established tool in DVA practice settings [23] documenting the presence, severity and escalation of four types of abuse (physical, sexual, harassment and stalking, jealous coercive and controlling behaviour). Responses to the presence of any type of abuse are indicated on a 3-point ordinal scale (standard, moderate, high). Severity and frequency are recorded on a 3-point ordinal scale (reduced, unchanged, worse). Participants own appraisal of safety was self-reported at Time 2 on a single-item scale reflecting ‘feelings of safety’ assessed on a 5-point ordinal scale (much safer, somewhat safer, no change, less safe). Cessation of abuse at exit was calculated by combining responses to the Severity of Abuse Grid into ‘Yes’ or ‘No’ categories to indicate the presence of ongoing abuse.

*Health outcomes dataset* included a sub-sample of hospital and community IDVA participants who provided information about their physical and mental health. Data were collected at two time points. Time 1 reflected intake IDVA service (T1 *N* = 114 hospital; *N* = 86 community). Time 2 data was collected 3 months after the participant had exited IDVA services (T2 *N* = 32 hospital; *N* = 4 community). Time 2 data were available for 36.0% of the hospital IDVA sub-sample and 4.6% of the community IDVA sub-sample. Owing to virtual absence of data from the community IDVA sub-sample, only findings from the hospital IDVA service were analysed and reported for health outcomes.

*SF12 Health Survey (Physical health composite score (PCS) and Mental health composite score) (MCS)* consists of 12 items designed to measure and monitor health [24]. The 12 items map onto components reflecting physical functioning, role limitations due to physical problems and emotional problems, mental health, energy/vitality and pain. Items measured on nominal / ordinal scales, are totalled to produce two mean scores of physical health (PCS12) and mental health (MCS12); where lower scores represent lower health. Time 2 data were available for 31.6% of the T1 hospital IDVA sample (T1 *n* = 101; T2 *n* = 32).

*HADS Hospital Anxiety and Depression Scale* [25] comprises 14 items assessed on a 4-point ordinal scale. Seven items are totalled to produce mean scores for the two component subscales measuring Anxiety and Depression. Scores of 0–7 suggest ‘normal’ levels of anxiety or depression, 8–10 borderline ‘abnormal’, 11–21 ‘abnormal’. Time 2 data were available for 32.9% of the T1 hospital IDVA sample (T1 *n* = 97; T2 *n* = 32).

*Primary Care Post-Traumatic Stress Disorder Screen* [26] is a 5-item measure that begins with an item designed to assess whether the participant has had any exposure to traumatic events over the course of their life. If the answer is ‘No’ to this initial item, the PC-PTSD-5 is completed with a score of 0. If the answer is ‘Yes’, five additional questions (yes, no) are asked about how that trauma exposure has affected participants over the past month. A score of 4+ indicates probable symptomology in-line with a diagnosis of post-traumatic stress disorder. Time 2 data were available for 35.6% of the T1 hospital IDVA sample (T1 *n* = 73; T2 *n* = 26).

*Health Service use* was recorded at T1 community (*n* = 76) and hospital IDVA participants (*n* = 38) and T2 hospital participants only (*n* = 31); participants were asked to recall their use of hospital services (inpatient, outpatient, ED and ambulance), local and mental health services and social care services over the previous 6 months or 6 months at T1 and T3 respectively.

### Analysis

We tested differences between hospital and community IDVA services, using the community IDVA data as the comparator to our hospital data. The results of t-tests and chi-square statistics are reported for comparison of demographic variables. Non-parametric tests were used to explore pre-and post-intervention outcomes (abuse frequency and severity, cessation of abuse, safety, health outcomes and health resource use) as the data was not normally distributed, and median values were reported in addition to mean values. Scores on the SF12 and HADS scales were compared to population figures for the UK [27, 28]. A multi-variate model was used to identify the factors associated with survivor’s appraisals of safety and cessation of abuse for IDVA services. Covariates correlated with ‘feelings of safety’ and ‘cessation of abuse’ were included in the models. Examination of variance inflation factor (VIF) and tolerance statistics indicated multicollinearity for the outcome ‘cessation of abuse’ but not for the model applied to ‘feelings of safety’. Consequently, ordinal and logistic regression techniques were used to identify factors which may contribute to the outcome variable ‘feelings of safety’. There were no differences in the two models, therefore logistic regression findings have been reported for ease of interpretation. This analysis was conducted on the total sample which included men and women. Typically, the experiences of men and women who experience DVA tend to be different [29]. Bivariate analyses of the regression models revealed no differences according to gender, potentially owing to the small number of male cases. A decision was taken to include men within the samples as one of the key research questions was to understand the difference in who was identified by hospital IDVA services compared to community IDVA services. Analyses dealt with missing data listwise which reduced the sample size dramatically across some variables particularly within the community IDVA sub-sample. This meant that some variables could not be included in the analyses (documented in tables), to this effect in addition to N’s ns’s have been reported. Health resource unit costs were compiled from NHS Reference Costs (2013/14) and Personal Social Services Research Unit (PSSRU) [30, 31] (Additional file 1). Health service use data in the 6 months pre IDVA intervention were compared for hospital and community survivors, generating a cost to services. Owing to a lack of data at T2 (6 months post intervention) T1 and T2 comparisons could only be made for hospital survivors. One survivor in the hospital IDVA group was considered an outlier as she alone counted for 29% of the ED visits and 40% of ambulance trips. Results of the cost analysis are reported for the entire sample and with exclusion of this outlier. This did not affect the difference in the overall health service use between hospital and community survivors. An estimate of potential cost savings generated by hospital IDVAs was performed and compared to the overall cost of setting up hospital IDVA services (Additional file 1).

## Results

### Characteristics of survivors accessing hospital IDVA services

Table 1 presents the demographic characteristics of survivors at the point of accessing hospital IDVA services (T1) compared to those working with community IDVA services. Most survivors supported by both services were white British or Irish (84.2% hospital; 77.5% community), heterosexual (98.0% hospital; 90.2% community) women (93.6% hospital; 96.2% community) who were, on average, in their mid-thirties (*M* = 35.6, 95% CI 34.6 to 35.4; hospital; *M* = 34.9, 95% CI 34.5 to 35.3 community). In both settings, smaller numbers of black, Asian, minority and ethnic groups (BAME) (15.3% hospital; 17.1% community), men (5.1% hospital; 4.2% community) and lesbian, gay, bisexual or transgender (LGBT) (2.0% hospital; 2.2% community) survivors were identified. Survivors working with hospital IDVAs were more likely to be pregnant (17.1% hospital; 6.3% community) or not have children at home (67.2% community; 51.1% hospital). A higher proportion of hospital survivors were aged over fifty-five (10.1% hospital; 6.8% community) and came from higher-income households (salaries over £36,400 per annum) (9.1% hospital; 4.2% community). Hospital survivors reported higher levels of complex needs and additional vulnerabilities including mental health difficulties (57.3% hospital; 35.2% community), alcohol (18.4% hospital; 8.3% community) and drug use problems (11.2% hospital; 5.2% community), financial difficulties (40.1%; hospital; 30.3% community) and disability (12.2% hospital; 8.3% community). Twice as many hospital survivors had ever planned or attempted suicide (36.3% hospital; 16.2% community) or had self-harmed (43.5% hospital; 23.5% community).

**Table 1:** Characteristics of survivors accessing hospital and community IDVA services at T1

	Hospital IDVA	Community IDVA
	%	%
Gender (*ns* = 677, 3506)		
Male	5.1	4.2
Female	93.6	96.2
Sexual orientation (*ns* = 678, 3527)		
Lesbian/gay/bisexual	2.0	2.2
Heterosexual	98.0	90.2
Age (*ns* = 686, 3529)		
*M* (SD) / [CI]	35.6 (13.1) [34.6, 35.4]	34.9 (11.8) [34.5, 35.3]
Over 55^a^	10.1**	6.8
Ethnicity (*ns* = 689, 3523)		
Black/minority ethnic	15.3	17.1
White/British/Irish	84.2	77.5
Relationship status (*ns* = 689, 3541)		
Current partner	53.4***	31.6
Ex-partner	35.3	59.7***
Children in the household (*n* = 692, 3544)		
Children at home	51.1	67.2***
Pregnant	17.1***	6.3
Household income (*ns* = 277, 1429)		
High £34,000+ p.a.	9.1***	4.2
Middle £33,999–15,599 p.a	15.1	12.4
Low 15,549 and below	49.4	55.3
Living arrangements (*ns* = 692, 3536)		
Cohabiting	48.3***	29.7
Living separately	43.4	62.2***
Survivors’ complex needs (*ns* = 667, 3474)		
Alcohol misuse	18.4***	8.3
Drug misuse	11.2***	5.2
Financial difficulties	40.1***	30.3
Disability	12.2***	8.3
Mental health	57.3***	35.2
Suicidal ideation / behaviours	36.3***	16.2
Self-harm	43.5***	23.5
Perpetrator information (*ns* = 686, 3545)		
Previously abusive to family or other partner	79.3***	67.7
Previous criminal record for domestic violence	36.6	45.2**
Multiple perpetrators	14.3***	8.3
Length of time survivor experienced abuse (months) (*n* = 692, 3544)		
*Mdn* (IQR)	30.0 (60.1)	36.3 (72)
Type of abuse experienced (*ns* = 683, 3537)		
Severe physical	46.6**	41.2
Severe sexual	14.3**	10.2
Severe controlling coercive behaviour	47.3	47.1
Severe harassment and stalking	30.8	34.1
Abuse that is escalating in severity or frequency	57.2	68.3*
At high risk (professional judgement)	53.1	58.2*

*** *p* < .001,**p < .01, *p* < .05*

Note: Over 55a. T-test revealed no significant difference in mean age. Owing to high variation in data, age was split across four age bandings according to the distribution (< 18 to 21, 21 to 40, 40 to 55, 55>) and coded into ordinal variables. Significance testing of data accordingly revealed differences in age for those over 55+ between hospital and community IDVA services

In the 3 months before accessing IDVA services, survivors in both settings experienced a high level of severe physical abuse (46.6% hospital; 41.2% community), jealous controlling and coercive behaviours (47.3%; hospital; 47.1% community) and harassment or stalking (30.8% hospital; 34.1% community). For all survivors, this abuse had escalated in severity and frequency within the last 3 months (57.2% hospital; 68.3% community). For community IDVA services, this escalation was higher and community IDVAs were more likely to deem their cases at higher risk of serious harm or fatality from the abuse (53.1% hospital; 58.2% community). However, hospital survivors were more likely to report severe forms of sexual abuse in the previous 3 months (14.3% hospital; 10.2% community). At the point of engaging with hospital IDVA services, survivors had experienced abuse for shorter periods (abuse length in months; *Mdn* = 30.0, IQR 60.1) compared with community services (*Mdn* = 36.3, IQR 70.0).

Community IDVAs tended to support survivors who were experiencing abuse from an ex-partner (35.3% hospital; 59.7% community). Hospital survivors were more likely to experience abuse from a current intimate partner (53.4% hospital; 31.6% community) or multiple perpetrators (14.3% hospital; 8.3% community). Higher proportions of hospital survivors were living with the abuser at the point of referral (48.3% hospital; 29.7% community). Despite being more likely to have been abusive to other partners or family members (79.3% hospital; 67.7% community), those perpetrating abuse towards hospital survivors were less likely to have a criminal record for DVA (36.6% hospital; 45.2% community).

### Identifying and referring survivors of domestic abuse across healthcare settings

Table 2 presents the help-seeking behaviours reported by hospital survivors compared with community survivors in the 6 months preceding support from an IDVA service. Those working with community IDVA services were more likely to have called the police (77.2%) than those supported by a hospital IDVA (58.7%); whereas, hospital survivors had accessed a greater number of health services for issues specifically related to DVA. They were more likely to have visited their GP (88.3% hospital; 77.2% community) and ED (56.2% hospital; 16% community). More hospital survivors had attended ED by ambulance (37.3% hospital; 16.3% community) than community survivors. Around a half (45.7%) of survivors identified by hospital IDVA services had done so after an overdose or because of mental ill health (50.6%); while 13.4% had visited ED because of physical injuries from the abuser.

**Table 2:** Help-seeking behaviours 6 months before accessing an IDVA service at T1

	Hospital IDVA	Community IDVA
	%	%
Help seeking (*ns* = 488, 3211)		
Saw GP for any reason	88.3***	77.2
Called the police	58.7	77.2***
Attended Accident and Emergency as a result of abuse	56.2***	16.3
Attended ED by ambulance as a result of the abuse	37.3**	16.2
Hospital IDVA survivors reasons for accessing ED (*n* = 103)^a^		
Visit because of physical injuries of abuser	13.4	–
Visit for mental health reason	50.6	–
Visit to ED after an overdose	45.7	–

*** *p* < .001, **p < .01, *p* < .05*

^a^Large amount of missing data for the community IDVA sample meant that comparative figures could not be reported for these indicators

Analysis of referral routes into the hospital IDVA service (Table 3) show that 84.6% of cases came from other health services, mostly within the hospital itself. ED played a key role in identifying survivors, accounting for over half (62.3%) of hospital IDVA referrals, followed by maternity and ante/neonatal units (16.8%) and psychiatry or mental health departments (7.3%). Nurses identified the greatest number of survivors (45.6%), followed by consultants/doctors/junior doctors (18.2%), midwives (13.7%) and psychiatrists/psychologists (8.4%). Comparatively, in community IDVA services, referrals were less likely to come from health services (2.3%). At the point of exiting the service, hospital IDVAs had helped survivors to access a higher number of health-based services than community IDVAs. Hospital survivors were more likely to have been referred to mental health services (22.9% hospital; 14.9%) and substance services (34.0% hospital; 3.3% community); whereas, community IDVAs referred higher proportions of survivors to the police (52.1% hospital; 83.7%).

**Table 3:** Referral routes into hospital and community IDVA services at intake (T1) and exit (T2)

	Hospital IDVA	Community IDVA
	%	%
Referral route to IDVA service T1 (*ns* = 283, 2430)		
Health	84.6***	2.3
Police	9.6	45.3***
Self	2.2	23.4***
Hospital department referrals to hospital IDVA T1 (*n* = 170)^a^		
ED	62.3	–
Maternity, ante- and neo-natal units	16.8	–
Psychiatry / mental health	7.3	–
Hospital staff referrals to hospital IDVA T1 (*n* = 164)^a^		
Nurse	45.6	–
Consultant/Doctor/Junior Doctor	18.2	–
Midwife	13.7	–
Psychologist/Psychiatrist	8.4	–
Ward Sister	4.5	–
Referral route out of IDVA service T2 (*ns* = 476, 2430)	52.1	83.7***
Police	66.0***	15.3
Housing	22.9*	14.9
Mental health	34.0**	3.3
Substance services	3.2	1.6
Adult social services		

*** *p* < .001,**p < .01, *p* < .05*

^a^This information was only collected for survivors accessing the hospital IDVA service

### Length and type of support

Support provided by hospital IDVAs (Table 4) comprised regular contact and access to a number of community programmes and resources. On average, hospital survivors were supported for just under 2 months (months *Mdn* = 1.7, IQR 2.7) which was shorter than support at community services (months *Mdn* = 2.4, IQR 3.1). However, hospital IDVAs worked as intensively as community IDVAs over this period with both services delivering the same number of face-to-face contacts with survivors (*Mdn* = 8.0 both hospital and community). Both IDVA services were most likely to provide support around safety planning, health and wellbeing, the police and housing. Survivors working with hospital IDVAs were more likely to have been helped to access safety planning (72.3% hospital; 63.4% community), health and wellbeing services (67.7% hospital; 56.3% community), the police (47.8% hospital; 41.2% community) and housing (45.3% hospital; 31.4% community). Hospital survivors were less likely than community IDVA survivors to have been helped to access civil orders (5.2% hospital; 14.3% community) or support with the criminal courts (1.1% hospital; 4.4% community).

**Table 4:** Support provided to survivors accessing hospital and community IDVA services at T2

	Hospital IDVA	Community IDVA
Case length (*N* = 521, 2259)		
*Mdn* (IQR) in months	1.7 (2.7)	2.4 (3.1)***
Frequency of contact with IDVA (*ns* = 533, 2376)		
*Mdn* (IQR)	8.0 (9.0)	8.0 (11.0)
% < 5 contacts	21.2	40.6
% > 5 contacts	78.4	60.3
Types of support accessed (*ns* = 692, 2722)		
% Safety planning	72.3***	63.4
% Health and wellbeing	67.7***	56.3
% Police	47.8*	41.2
% Housing	45.3***	31.4
% Marac	34.4	32.3
% Children	24.5	24.6
% Finance / benefits	17.2*	13.2
% Civil orders	5.2	14.3***
% Probation	3.3	5.1
% Criminal court	1.1	4.4***

*** *p* < .001, **p < .01, *p* < .05*

### Health outcomes

Health measures at the point of accessing a service (T1) demonstrated that hospital IDVAs worked with survivors who reported poorer physical health (T1 *M* = 49.2, 95% CI 47.1 to 53.9) and substantially poorer mental health (T1 *M* = 32.3, 95% CI 29.5 to 34.2) compared with the general UK population (Table 5). Among hospital.

**Table 5:** Health outcomes for survivors accessing hospital IDVA services T1 compared with T2

	Time one (T1)		Time two (T2)		UK population	
Health measures hospital IDVA sample (*n* = 64, 21)	*M*	SD / [95% CI]	*M*	SD / [95% CI]	*M*	SD
Physical health (SF12-PCS)	49.2	12.8 [47.1, 53.9]	48.7	11.4 [45.9, 54.0]	50.9	9.4
Mental health (SF12 - MCS)	32.3	12.2 [29.5, 34.2]	39.6	12.8 [34.9, 44.3]	52.1	8.7
Anxiety (HADS - Anxiety)	12.2	5.1 [10.6,13.5]	11.4	6.3 [10.6,12.6]	6.7^a^	4.2
Depression (HADS - Depression)	10.5	5.2 [7.9, 12.2]	8.6	6.1 [7.9, 10.0]	5.5*	4.0
Post-Traumatic Stress Disorder (PTSD)	%		%			
	62.6		48.6			
*M* (SD) / [95% CI]	2.1	1.65 [1.7, 2.5]	2.0	1.5 [1.4, 2.6]		

^a^ UK normative data for females [56]. Women score higher on anxiety scales, no significant differences in depression between men and women

Note. No statistical differences were found between T1 and T2 for health outcomes; all measures = *p* < NS

survivors, levels of anxiety (T1 *M* = 12.2, 95% CI 10.6 to 13.5) and depression (T1 *M* = 10.5, 95% CI 7.9 to 12.2) were twice the national average. At T1, over half (62.6%) screened positive for Post-Traumatic Stress Disorder (PTSD) (T1 *M* = 2.1, 95% CI 1.7 to 2.5). Between T1 and T2 (3 months after exiting support for DVA), no changes were observed in health outcomes among hospital survivors. While survivors reported a lower level of physical (T2 *M* = 48.7, 95% CI 45.9 to 54.0) and mental health concerns at T2 (T2 *M* = 39.6, 95% CI 34.9 to 44.3), specifically around anxiety (T2 *M* = 11.4, 95% CI 10.6 to 12.6), depression (T2 *M* = 8.6, 95% CI 7.9 to 10.0) and PTSD symptomology (T2 *M* = 2.0, 95% CI 1.4 to 2.6), no significant differences were observed; potentially owing to the small sample size.

### DVA outcomes

Outcomes relating to the change in DVA assessed at the closure of cases revealed some positive changes in safety for survivors accessing both IDVA services (Table 6). Survivors accessing hospital IDVA services were more likely to experience cessation of abuse at the point of exiting the service than survivors identified by community IDVA services (62.4% hospital; 48.3% community). Hospital survivors reported a higher level of reduction in physical abuse (86.2% hospital; 71.2% community), sexual abuse (82.4% hospital; 73.3% community), harassment and stalking (75.6% hospital; 52.4% community) and jealous coercive and controlling behaviours (70.1% hospital; 52.2% community). Hospital survivors were more likely to report that they felt ‘much safer’ (54.2%) compared to survivors who accessed a community service (50.1%). Across both services, several survivors reported a continuation of abuse at exit. Abuse was ongoing in 10.2% of hospital IDVA cases and 18.4% of community IDVA cases.

**Table 6:** DVA outcomes and reductions in abuse for survivors accessing hospital and community IDVA services

	Hospital IDVA		Community IDVA	
DVA outcomes at T2	%		%	
Cessation of abuse (*ns* = 476, 2722)				
% ceased	62.4**		48.3	
% ongoing	10.2		18.4	
% don’t’ know / missing	27.4		33.3	
Reductions in reported abuse				
% reporting physical abuse	86.2**		71.2	
% reporting sexual abuse	82.4*		73.3	
% reporting harassment and stalking	75.6**		52.4	
% reporting jealous coercive and controlling behaviour	70.1*		52.2	
Survivor appraisal of safety				
% much safer than before	54.2*		50.1	
% somewhat safer than before	30.1		36.4	
% no safer than before	7.2		9.1	
% don’t know/missing	8.5		4.4	
Reductions in abuse between T1 & T2				
Report of abuse between intake and exit (ns = 476, 2722)	T1	T2	T1	T2
% physical abuse	70.3	10.2***	60.3	18.4**
% sexual abuse	25.4	5.3***	19.2	5.8***
% harassment and stalking	66.6	17.4**	62.2	30.1*
% jealous coercive and controlling behaviour	87.5	18.5*	85.4	40.2*

*** *p* < .001,**p < .01, *p* < .05*

Table 7 presents the results of logistic regression analyses examining the association between the different resources / programmes received and reported safety among survivors who accessed IDVA services, controlling for potentially confounding variables. Analyses showed that safety increased if the support provided was more intensive. Survivors who had accessed a hospital IDVA service were two times more likely to report feeling safer at case closure (AOR = 2.03, 95% CI 1.18 to 3.49) if they had received over five or more contacts with an IDVA. Similarly, survivors accessing the hospital IDVA service were found to have higher odds of achieving feelings of safety if they had been supported over a longer period and had accessed a higher number of resources / programmes provided by wider community services. Accessing six or more resources / programmes increased safety by one and a half times AOR = 2.38, 95% CI 1.41 to 3.87) and odds of achieving this outcome increased progressively with a greater number of support days provided by the IDVA (AOR = 2.00, 95% CI 1.00 to 1.01). Survivors who had accessed a hospital IDVA service were more likely to report no change or feeling less safe at exit if they had experienced suicidal ideation or behaviours at the point of initial referral (AOR = 2.00, 95% CI 0.28 to 0.74). The same model was applied to the community IDVA cases and findings were replicated, whereby, feelings of safety were increased in line with more intensive support in terms of more frequent contact with a community IDVA (AOR = 1.45, 95% CI 1.12 to 1.89) and access to a range of resources / programmes (AOR = 1.82, 95% CI = 1.43 to 2.31).

**Table 7:** Factors influencing feelings of safety after accessing hospital and community IDVA services at T2

	Hospital IDVA - Survivor felt safer (1) vs not safer/missing (0)								Community IDVA - Survivor felt safer (1) vs not safer/missing (0)							
							95% C.I.for EXP(B)								95% C.I.for EXP(B)	
	B	S.E.	Wald	df	Sig.	AOR	Lower	Upper	B	S.E.	Wald	df	Sig.	AOR	Lower	Upper
6+ interventions accessed (1) vs 0–1 accessed (0)	.84	.25	10.8	1	.001	2.3	1.4	3.8	.59	.12	23.903	1	.000	1.8	1.4	2.3
5+ contacts with IDVA (1) vs < 5 (0)	.71	.27	6.6	1	.010	2.0	1.2	3.4	.37	.13	8.034	1	.005	1.4	1.1	1.8
Length of case (days)	.00	.00	7.0	1	.008	1.0	1.0	1.0	.00	.00	.011	1	.917	1.0	.99	1.0
Reported suicidal ideation/behaviour T1 (1) vs none recorded (0)	−.77	.24	10.2	1	.001	.46	.28	.74	−.19	.15	1.481	1	.224	.82	.60	1.1
Any abuse escalating in severity and frequency at T1 (1) vs none recorded (0)	−.36	.29	1.5	1	.219	.69	.38	1.2	.20	.13	2.143	1	.143	1.2	.93	1.5
Physical abuse at T1 (1) vs none recorded (0)	−.07	.27	.08	1	.775	.92	.53	1.5	−.12	.12	.913	1	.339	.88	.68	1.1
Harassment and stalking at T1 (1) vs none recorded (0)	−.08	.25	.10	1	.752	.92	.56	1.5	−.00	.11	.002	1	.968	.99	.78	1.2
Jealous coercive and controlling behaviour at T1 (1) vs none recorded (0)	.11	.36	.10	1	.750	1.1	.54	2.3	−.10	.16	.388	1	.533	.90	.66	1.2
Drug/alcohol misuse at T1 (1) vs none recorded (0)	−.04	.32	.02	1	.897	.95	.50	1.8	−.05	.22	.049	1	.825	.95	.60	1.4
Mental health issues at T1 (1) vs none recorded (0)	−.48	.36	1.7	1	.186	.61	.29	1.2	−.51	.25	4.023	1	.045	.59	.36	.98
Perpetrator drug/alcohol misuse at T1 (1) vs none recorded (0)	−.39	.37	1.1	1	.292	.67	.32	1.3	.15	.18	.769	1	.381	1.1	.82	1.6
Perpetrator mental health at T1 (1) vs none recorded (0)	.36	.40	.81	1	.368	1.4	.65	3.1	.09	.18	.268	1	.605	1.1	.76	1.5
Perpetrator financial issues at T1 (1) vs none recorded (0)	−.33	.40	.68	1	.408	.71	.32	1.5	−.05	.18	.073	1	.787	.95	.66	1.3
Constant	.41	.43	.93	1	.333	1.5			.94	.20	22.2	1	.000	2.5		

*Hospital n* = 451; Model statistics: -2LL = 471.46, X^2^ = 57.06, df = 13, *p <* .001, Nagelkerke *R*^2^ = .17, % classified correctly = 75%

*Community n* = 2177; Model statistics: -2LL = 1877.80, X^2^ = 142.96, df = 13, *p <* .001, Nagelkerke *R*^2^ = .16, % classified correctly = 81%

### Health resource use and cost analysis

In the 6 months before accessing IDVA services, hospital survivors used more health services than community survivors (Table 8). In terms of single components, differences were observed for general practices (hospital *M* = 4.9, 95% CI 3.7 to 6.0; community *M* = 2.6, 95% CI 1.8 to 3.9), mental health services (counsellors) (hospital *M* = 3.0, 95% CI 0.4 to 6.4; community *M* = 1.5, 95% CI 0.0 to 3.0), inpatient stays (hospital *M* = 3.6, 95% CI 1.2 to 6.0; community *M* = 0.3, 95% CI 0.0 to 0.7), ED attendance (hospital *M* = 1.0, 95% CI 0.6 to 1.3; community *M* = 0.4, 95% CI 0.1 to 0.5) and ambulance trips (hospital *M* = 0.6, 95% CI 0.3 to 0.1; community *M* = 0.2, 95% CI 0.0 to 0.3). The cost analysis based on health resource use showed that hospital survivors cost on average £2248 (95% CI £1646 to £2977) and community survivors cost on average £533 (95% CI £373 to £713).

**Table 8:** Health resource use at T1 compared with T2 (hospital survivors only)

	Hospital survivors *n* = 76			Community survivors *n* = 38			Hospital survivors T1 *n* = 29			Hospital survivors T2 *n* = 29			
Health resource	*M* use	SD / 95% CI	*M* cost	*M* use	SD / 95% CI	*M* cost	*M* use	SD / 95% CI	*M* cost	*M* use	SD / 95% CI	*M* cost	Diff. T1 - T2
GP surgery consultation	4.9**	4.9 [3.7, 6.0]	239	2.9	3.2 [1.8, 3.9]	142	4.0	4.3 [2.5, 5.8]	198	5.3	4.9 [3.4, 7.2]	261	-£63
GP phone consultation	1.3	4.2 [0.3, 2.3]	27	0.5	1.2 [0.1, 0.9]	10	1.8	5.6 [0.2, 4.0]	37	1.2	2.2 [0.3, 2.0]	24	£13
Practice Nurse consultation	2.0	7.4 [0.2, 3.6]	24	0.7	1.5 [0.3, 1.2]	9	1.5	4.8 [0.2, 3.4]	19	1.1	2.0 [0.3, 1.9]	14	£5
Community Psychiatric Nurse	0.9	2.8 [0.2, 1.5]	33	0.0	0.2 [0.03, 0.8]	1	0.7	2.7 [0.3, 1.8]	28	1.7	5.4 [0.3, 3.6]	63	-£35
Psychiatrist	0.4	1.2 [0.1, 0.7]	45	0.0	0.2 [0.03, 0.8]	3	0.5	1.2 [0.0, 0.9]	50	0.9	4.3 [0.7, 2.5]	100	-£50
Clinical Psychologist	0.8	3.8 [0.1, 1.6]	160	–	–	–	1.5	5.9 [0.7, 3.8]	325	1.7	5.5 [0.4, 3.7]	353	-£28
Health Visitor	2.0	9.2 [0.2, 4.9]	106	1.0	3.0 [0.3, 1.9]	53	0.7	1.9 [0.0, 1.5]	38	0.5	1.8 [0.2, 1.1]	25	£13
Counsellor	3.0**	14.6 [0.4, 6.4]	138	1.5	4.6 [0.0, 3.0]	69	2.0	6.8 [0.5, 4.6]	92	2.1	5.3 [0.1, 4.1]	98	-£6
Psychotherapist	0.1	0.9 [0.1, 0.3]	17	0.0	–	–	0.3	1.4 [0.3, 0.8]	42	0.0	–	0	£42
Family therapist	0.0	–	–	0.1	0.3 [0.0, 0.1]	8	0.0	–	0	0.1	0.7 [0.1, 0.4]	21	-£21
Drug/alcohol support	0.9	3.5 [0.0, 1.6]	66	0.2	0.7 [0.0, 0.4]	12	1.1	3.2 [0.1, 2.3]	83	1.5	5.0 [0.4, 3.4]	120	-£37
In-patient stay per night	3.6**	10.3 [1.2, 6.0]	997	0.3**	1.2 [0.0, 0.7]	94	4.5	14.2 [0.8, 10.0]	1238	0.0**	–	0	£1238
Outpatient appointments	1.3	2.4 [0.7, 1.7]	142	0.4	1.2 [0.0, 0.8]	50	0.8	1.9 [0.3, 1.1]	93	2.7	6.2 [0.3, 5.0]	296	-£203
A&E attendance	1.0**	1.4 [0.6, 1.3]	118	0.4	0.6 [0.1, 0.5]	46	0.9	1.4 [0.3, 1.4]	107	0.4	0.9 [0.0, 0.7]	50	£57
Ambulance trip	0.6*	1.3 [0.3, 0.1]	136	0.2*	0.4 [0.0, 0.3]	36	0.6	1.4 [0.0, 1.0]	131	0.1	0.5 [0.0, 0.3]	31	£100
Total (95% CI)		£2248 (£1646 to £2977)			£533 (£373 to £713)			£2481 (£1731 to £3629)			£1456 (£192 to £2063		£1025 (£182 to £2030)

*** *p* < .001,**p < .01, *p* < .05*

Based on the difference in resource use 6 months before (T1) and 6 months after (T2) accessing the hospital IDVA service, a cost reduction was observed post-intervention in most hospital services. Hospital survivors reported fewer inpatient stays (T1 *M* = 4.5, 95% CI 0.8 to 10.0; T2 *M* = 0.0), ED attendances (T1 *M* = 0.9, 95% CI 0.3 to 1.4; T2 *M* = 0.4, 95% CI 0.0 to 0.7) and ambulance trips (T1 *M* = 0.6, 95% CI 0.0 to 1.0; T2 *M* = 0.1, 95% CI 0.0 to 0.3). Outpatient appointments increased pre and post intervention (T1 *M* = 0.8, 95% CI 0.3 to 1.1; T2 *M* = 2.7, 95% CI 0.3 to 5.0). Decreased use of hospital services offset the increase in cost observed in slightly higher levels of attendance at general practices (T1 *M* = 4.0, 95% CI 2.5 to 5.8; T2 *M* = 5.3, 95% CI 3.4 to 7.2), mental health services (community psychiatric nurse) (T1 *M* = 4.0, 95% CI 2.5 to 5.8; T2 *M* = 5.3, 95% CI 3.4 to 7.2) and substance services (T1 *M* = 1.1, 95% CI 0.1 to 2.3; T2 *M* = 1.5, 95% CI 0.4 to 3.4). However, the only significant difference in health service use pre and post intervention was attributed to the decrease in hospital inpatient stays; potentially owing to the small sample size. Overall, the cost reduction post intervention was equivalent to savings of £2050 per patient per year when the resource use is extrapolated to a one-year period (6 months £1025, 95% CI £182 to £2030).

## Discussion

This article reports findings from a multi-site evaluation of a healthcare-based advocacy intervention which co-located IDVAs in five UK hospitals. Results highlight the advantages of placing IDVAs within ED and maternity departments compared to community IDVA services.

### Earlier intervention and support for ‘hidden’ survivors

Hospital IDVA services enabled access to support for survivors less visible to community IDVA services. Although referrals for some such survivors -- BAME people, LGBT people, and men -- were low, hospital IDVAs worked with survivors who were older (aged over 55), from higher income households and who were pregnant. This mirrors previous research [20, 21]. Our findings highlight that hospital IDVAs were more likely to engage with survivors at an earlier point in the abusive relationship, often when survivor and perpetrator were still living together. This demonstrates hospital IDVA services may provide an opportunity for earlier intervention.

### Integration within the hospital community

In line with previous research, survivors accessing hospital IDVA services were more likely to see their GP and attend ED for issues related to DVA in the 6 months preceding support but were less likely to call the police [7, 8]. The number of survivors presenting to ED particularly by ambulance demonstrates the need for paramedics and ED healthcare professionals to be trained in effectively responding to DVA. A key component of the hospital IDVA role was to raise awareness of DVA services within the hospital community and provide training to healthcare professionals. The analysis of referral routes into IDVA services shows the advantages of this aspect of the service, with increased referrals into the hospital IDVA from a range of hospital departments. Similarly, hospital IDVAs enabled a greater number of survivors to access ongoing health services (e.g., mental health and substance services) at the point of case closure. Reflecting existing research, this suggests that the co-location of IDVAs in hospitals provides clear referral pathways for survivors to specialist DVA support that is immediately accessible but also other health services for longer term care [19–21].

### Outcomes for survivors

By case closure, most survivors reported reductions in all forms of abuse and over half felt safer. Survivors engaging with hospital IDVA services were more likely to experience cessation of abuse and greater reductions in abuse from T1 to T2 than survivors accessing community IDVA services. However, within the community IDVA service abuse tended to be more severe at intake, potentially leading to lower reductions in non-physical forms of abuse at the point of exit. In line with previous research, intensive support from IDVAs (five or more contacts) over a longer period and access to a greater number of community resources / programmes increased the likelihood of safety [17, 18]. These results demonstrate the importance of hospital-IDVAs working as part of a co-ordinated multi-agency response.

No changes were observed in health outcomes for survivors who had accessed the hospital IDVA. These findings may reflect the small size of the sample or the short time period at which health outcomes were assessed after case closure. However, 3 months after accessing support, reported levels of health were still proportionately lower than UK averages. Survivors accessing hospital IDVA services experienced diminished odds of feeling safe if at the point of intake they were experiencing suicidal thoughts / behaviours. Given the high proportion of mental health issues among this population, the continued presence of mental health services is particularly vital.

### Financial implications of hospital IDVA

The cost of providing hospital IDVA services may be ~ £315–£417 per survivor per year (Additional file 1). When compared to the potential cost savings associated with the intervention, providing hospital IDVA services would be cost-neutral for the NHS. For hospitals themselves, the intervention could be particularly cost-effective as the main reduction in costs after the intervention was entirely associated with hospital services.

### Limitations

There were several limitations relating to the evaluation design and data quality. The design was nonexperimental, which may have resulted in the overstatement of intervention effectiveness [32]. The DVA outcome measure (feelings of safety) was a self-reported single-item and included no measure of attribution. Owing to high levels of attrition at T2 determining health outcomes for survivors accessing community IDVA services was not possible. The cost analysis was limited as the resource use estimates were based on patient recollection. The sample size was limited, especially the hospital participants sample informing the pre- and post-intervention cost analysis. The analysis did not attempt to quantify the cost-effectiveness of the intervention due to the limited data available. Further research is required to support the efficacy of this intervention, ideally adopting an experimental design, using robust and validated outcome measures of DVA, with cost-effectiveness and health outcomes assessed over an extended time frame.

## Conclusion

Healthcare based interventions represent a specific approach to identifying, preventing and managing DVA in a location that survivors are likely to visit due to the range of health impacts that accompany experiences of abuse. Findings discussed here indicate that co-locating specialist domestic violence advocacy services (IDVAs) in hospitals can help healthcare professionals to identify abuse among their patients with the option to refer immediately for support. The advantages of locating IDVAs in hospitals include greater visibility of ‘hidden survivors’, an opportunity to intervene earlier and increased referrals from health services. Intensive support and access to community interventions were shown to have a positive impact on DVA outcomes. This study has shown that hospital IDVAs offer a unique and promising strategy for tacking DVA from within the healthcare service.

## Supplementary information

**Additional file 1.** Health resource unit costs compiled from NHS Reference Costs (2013/14), Personal Social Services Research Unit (PSSRU) [30, 31] and delivery costs for Hospital IDVA services provided by two sites included within the evaluation.

## Data Availability

The datasets generated and/or analysed during the current study are not publicly available because consent was not sought for this purpose. Data may be available from the corresponding author on request.

## Abbreviations

DVA: Domestic violence and abuse
GUM: Genitourinary medicine
HIV: Human immunodeficiency virus
IDVA: Independent domestic violence advisor
IPV: Intimate partner violence
NICE: National Institute of Health and Care Excellence
PTSD: Post-Traumatic Stress Disorder
UK: United Kingdom
WHO: World Health Organisation
