# Supplementary material for: Cry for health: a quantitative evaluation of a hospital-based advocacy intervention for domestic violence and abuse
Source: BMC Health Serv Res. 2019 Oct 21;19:718. doi: 10.1186/s12913-019-4621-0 (PMC6805459; doi:10.1186/s12913-019-4621-0)
Supplement: Supplementary file 1 — Additional file 1. Health resource unit costs compiled from NHS Reference Costs (2013/14), Personal Social Services Research Unit (PSSRU) [30, 31] and delivery costs for Hospital IDVA services provided by two sites included within the evaluation. [file 12913_2019_4621_MOESM1_ESM.docx]

| **Additional file 1. Health resource unit costs** | | |
| --- | --- | --- |
| **Healthcare resource** | **Cost** | **Source / Notes** |
| GP surgery consultation | £49.02 | ^(59)^ |
| GP home visit | £32.49 | ^(59)^ |
| GP phone consultation | £20.23 | ^(59)^ |
| Practice Nurse consultation | £12.14 | Based on the cost per hour of face to face contact (£47) and average consultation time of 15.5 minutes ^(59)^ |
| Community Psychiatric Nurse | £38 | Average cost of face to face contact in district nursing services ^(59)^ |
| Psychiatrist | £107 | Based on the cost per hour (£107) and assumption of one hour for duration of consultation ^(59)^ |
| Clinical Psychologist | £212 | Cost of consultant-led outpatient attendance for clinical psychology ^(60)^ |
| Health Visitor | £54 | Average cost of face to face contact in health visiting services ^(59)^ |
| Counsellor | £45.83 | Based on the cost per hour of counselling services in primary care (£55) and average consultation time of 55 minutes ^(59)^ |
| Psychotherapist | £156 | Cost of consultant-led outpatient attendance for clinical psychotherapy ^(59)^ |
| Family therapist | £156 | Same as psychotherapist (assumption) ^(59)^ |
| Drug/alcohol support | £78 | Addition services attendance ^(60)^ |
| In-patient stay per night | £275 | Mean cost per bed day ^(60)^ |
| Outpatient appointments | £111 | Mean cost of all outpatient attendances ^(60)^ |
| A&E attendance | £124 | Weighted average of all emergency medicine attendances ^(60)^ |
| Ambulance trip | £231 | Cost for see and treat and convey (currency code ASS02) ^(60)^ |
| Social worker/Child and Family support worker | £53 | Based on the average between cost per hour of a social worker (£55) and family support worker (£51). Assumption of one hour for duration of consultation. ^(60)^ |
| Hospital IDVA services | £315 to £417 | One hospital IDVA service cost £40,000 p.a. in staffing and £720 in clinical supervision (2015/16) for the equivalent of one full-time IDVA who worked with 97 cases during the year. This averaged a cost of £417 in IDVA service per client. Another hospital IDVA service cost £90,000 a year (including clinical supervision, publicity materials) for two full-time staff, who worked with 286 cases a year (2015-16). This averaged at a cost of £315 per client. This may be lower than the other service because there was a shorter, six-week time limit on cases, after which cases had to be passed onto local domestic abuse services. Cost information provided by two IDVA services in the study. |
